# The role of genetics in inherited cardiac diseases

**DOI:** 10.1515/medgen-2025-2004

**Published:** 2025-04-08

**Authors:** Gudrun Rappold, Bernd Wollnik

**Affiliations:** Heidelberg University Hospital Institute of Human Genetics Im Neuenheimer Feld 366 69120 Heidelberg Germany; Georg-August University Göttingen Institute of Human Genetics Heinrich-Düker-Weg 12 37073 Göttingen Germany

During the last decade, major progress has been made in understanding the genomic architecture and molecular mechanisms of cardiovascular diseases. Genetics in particular has progressed this field enormously, contributing to the extensive growth in genomic medicine. We have harnessed human genomic science for biological discovery, new therapeutic targets and genomic medicine. Also, we have gained new insights into cardiac biology and how to prevent and treat heart diseases. And the role of genetic diagnostics and genetic counselling has been recognized to be central to the practice of advanced genomic medicine.

Cardiogenetics in general studies the role of genetic factors contributing to cardiovascular diseases. In the case of monogenic cardiac diseases, pathophysiological changes and clinical symptoms are directly caused by the genetic variation(s). In the case of oligogenic or polygenic cardiac diseases, multiple genetic variants with low individual impact sum up to a collective effect. Large-scale GWAS have forwarded several hundreds of highly compelling loci across cardiovascular diseases and associated phenotypic traits, and polygenic risk scores are adding those variants up to estimate individual risk. Multi-centres registry studies and large biobank data sets furthermore are currently facilitating tremendous opportunities in genomics research.

We have also recently learned that identified gene variants can be disease-causing, predisposing, neutral or disease-protecting, that there can be tremendous inter- and intrafamilial variability of phenotypic expression, variable penetrance and additional pleiotropy meaning that some of the affected genes playing a role in cardiac diseases are also playing a role in other organ systems. The next important steps in cardiobiology will now use multi-omics approaches to link genomics, transcriptomics, proteomics, and metabolomics to the human phenome in order to completely understand the relation between molecular alterations and phenotypic outcome, at scale.

From today’s perspective, however, it is important to know which kind of genetic diagnostics are already in place and useful in practice. Chiara Vey, Nico Melnik, Gregor Dombrowsky, and Marc-Phillip Hitz review current diagnostics for **congenital heart disease** and discuss future options. They highlight the enormous variability of genetic causes, ranging from aneuploidies to single gene variants and complex inheritance patterns leading to syndromic and non-syndromic forms of early congenital heart disease. Their overall assessment is that genetic analysis is essential for predicting prognosis and recurrence risk in families with congenital heart disease and for guiding individual treatment options.

The contribution of Göhan Yigit, Silke Kaulfuß and Bernd Wollnik focusses on the clinical aspects and genetic determinants of inherited** cardiomyopathies**. To date, more than 100 genes are associated with cardiomyopathies, which can be divided into at least five distinct subtypes that overlap both clinically and genetically. Among these, variants in the titin (*TTN*) gene are the most common, causative variants in individuals with dilated non-syndromic cardiomyopathy. This gene, which has a large number of different isoforms, is used as an example to illustrate the challenges of classifying and interpreting identified variants in diagnostics. Another example of newly identified genes is *RPL3L*, which is associated with autosomal recessive childhood-onset cardiomyopathy, while heterozygous *RPL3L* variants elevate risk of atrial fibrillation.

Even a well-known and defined clinical syndrome such as **Noonan syndrome**, as highlighted by Martin Zenker and Cordula Wolf, leaves room for genetic challenges and clinical complexities. Noonan syndrome is considered a prototypical disease within the RASopathies, a group of pathogenetically related disorders with cardiovascular involvement. Clinically, the most prevalent congenital heart defects in Noonan syndrome at birth are pulmonary valve stenosis and septal defects, while hypertrophic cardiomyopathy with or without arrhythmia and abnormalities of the lymphatic system can manifest at various ages. So far almost 20 RASopathy-associated genes are included in diagnostic cardiomyopathy panels and the related RAS-MAPK and PI3K-AKT-mTOR pathways have significant roles during embryonic stages in RASopathies.

Cardiac arrhythmias are reviewed by Sven Dittmann, Janis Kerkering and Eric Schulze-Bahr. **Inherited arrhythmias** are genetically highly heterogeneous. While they are mostly associated with cardiac ion channels causing ion channel dysfunction, related regulatory units such as transcription factors and modifiers also play a role. Examples provided include genes associated with congenital long QT syndrome (11 genes), Brugada syndrome (7 genes), catecholaminergic polymorphic ventricular tachycardia (8 genes) and short QT syndrome (5 genes). Recommendations for genetic testing of specific inherited arrhythmias are provided and challenges of variant interpretation in the clinical setting discussed.

Disease mechanisms and novel **drug therapies for atrial fibrillation**, the most common multifactorial cardiac arrhythmia, are reviewed by Felix Wiedmann and Constanze Schmidt. Antiarrhythmic drugs and catheter ablation are the traditional treatments, while novel approaches aim to tailor gene-specific antiarrhythmic drugs. The potassium channel TASK-1 (*KCNK3*) is used as an example for the development of a gene-specific therapeutic intervention. While the pathophysiology of genes associated with atrial fibrillation focuses on ion channel dysfunction, calcium handling, oxidative stress and fibrosis, other novel genes in key pathways represent cardiac structural genes or transcriptional regulators. An overview of current and emerging approaches to the treatment of atrial fibrillation is provided.

The final chapter by Timon Seeger and Sandra Hoffmann focuses on patient-specific disease modelling using **human induced pluripotent stem cells (hiPSCs)**, which are often coupled with CRISPR-based genome editing. These cells have two key properties: the ability to self-renew and to differentiate into any cell type. Together with advances in cardiac tissue engineering into cardiac organoids and microtissues, differently engineered cardiac tissues and 3D-bioprinted cardiac tissues – these exciting developments will allow more accurate modelling of cardiac diseases and future therapeutic applications towards personalised gene and cell therapies.

With this compilation of exciting up-to-date reviews, we wish our readers a stimulating read.

